# Cationic Amino Acid Transporter-2 Regulates Immunity by Modulating Arginase Activity

**DOI:** 10.1371/journal.ppat.1000023

**Published:** 2008-03-14

**Authors:** Robert W. Thompson, John T. Pesce, Thirumalai Ramalingam, Mark S. Wilson, Sandy White, Allen W. Cheever, Stacy M. Ricklefs, Stephen F. Porcella, Lili Li, Lesley G. Ellies, Thomas A. Wynn

**Affiliations:** 1 Immunopathogenesis Section, Laboratory of Parasitic Diseases, National Institute of Allergy and Infectious Diseases, National Institutes of Health, Bethesda, Maryland, United States of America; 2 Biomedical Research Institute, Rockville, Maryland, United States of America; 3 Genomics Unit, Research Technologies Section, Rocky Mountain Laboratories, Hamilton, Montana, United States of America; 4 Centocor Inc., Malvern, Pennsylvania, United States of America; 5 Moores UCSD Cancer Center, University of California San Diego, La Jolla, California United States of America; University of Pennsylvania, United States of America

## Abstract

Cationic amino acid transporters (CAT) are important regulators of NOS2 and ARG1 activity because they regulate L-arginine availability. However, their role in the development of Th1/Th2 effector functions following infection has not been investigated. Here we dissect the function of CAT2 by studying two infectious disease models characterized by the development of polarized Th1 or Th2-type responses. We show that CAT2^−/−^ mice are significantly more susceptible to the Th1-inducing pathogen *Toxoplasma gondii*. Although *T. gondii* infected CAT2^−/−^ mice developed stronger IFN-γ responses, nitric oxide (NO) production was significantly impaired, which contributed to their enhanced susceptibility. In contrast, CAT2^−/−^ mice infected with the Th2-inducing pathogen *Schistosoma mansoni* displayed no change in susceptibility to infection, although they succumbed to schistosomiasis at an accelerated rate. Granuloma formation and fibrosis, pathological features regulated by Th2 cytokines, were also exacerbated even though their Th2 response was reduced. Finally, while IL-13 blockade was highly efficacious in wild-type mice, the development of fibrosis in CAT2^−/−^ mice was largely IL-13-independent. Instead, the exacerbated pathology was associated with increased arginase activity in fibroblasts and alternatively activated macrophages, both in vitro and in vivo. Thus, by controlling NOS2 and arginase activity, CAT2 functions as a potent regulator of immunity.

## Author Summary

Recent studies conducted with amino transporter *Slc7a2*-deficient mice (CAT2) demonstrated that NOS2 activity in macrophages is regulated by CAT2. NOS2, which synthesizes nitric oxide, regulates numerous important activities, including resistance to infectious organisms, tumor development, and autoimmune diseases. It also competes with the enzyme Arginase-1 (Arg1) for the common substrate L-arginine. However, the role CAT2 in the regulation of Arg1 activity has not been previously examined. Therefore, we infected CAT2-deficient mice with the helminth parasite *Schistosoma mansoni* or with the protozoan pathogen *Toxoplasma gondii*, two organisms that trigger highly divergent host immune responses. Strikingly, following infection with *S. mansoni*, CAT2^−/−^ mice developed parasite egg–induced lesions in the liver that were 3 to 4 times larger than wild type and hepatic fibrosis (a feature of severe schistosomiasis) was exacerbated, indicating a general worsening of disease in the absence of CAT2. The CAT2^−/−^ mice were also more susceptible to *T. gondii* infection, demonstrating that CAT2 is critical for the development of protective cell-mediated immunity. Thus, these studies identify CAT2 as a powerful regulator of host immune responses, which may have major implications for a variety of infectious, inflammatory, and autoimmune diseases.

## Introduction

Tissue macrophages comprise a heterogeneous population of cells, recently separated into three major categories based on their unique functional capabilities. The T_H_2 cytokines IL-4 and IL-13 trigger a characteristic ‘alternative’ state of activation in macrophages that is distinct from the ‘classical’ T_H_1-type activation by IFN-γ or deactivation phenotype associated with IL-10 and TGF-β [1]. In contrast to classically activated macrophages (CAMø), which regulate cellular immunity to intracellular pathogens, alternatively-activated macrophages (AAMø) are associated with chronic helminth infections and allergic disease [2],[3],[4]. AAMø's participate in humoral immune responses, facilitate clearance and presentation of antigens, and regulate the important process of tissue repair [1],[5].

In the murine model of schistosomiasis, mice chronically infected with *Schistosoma mansoni* develop severe liver pathology characterized by the formation of eosinophil-rich granulomas and fibrosis, which leads to portal hypertension, bleeding from collateral vessels, and ultimately death [6]. As with many helminth infections the immune response to *S. mansoni* is T_H_2-biased [7]. Consequently, AAMø's are the major macrophage subpopulation observed in schistosomiasis [8],[9], with recent studies suggesting their development is critical to the long-term survival of the infected host [10]. Although the exact role of AAMø's in inflammation and fibrosis remains unclear, numerous studies including our own have suggested they are important regulators of wound healing. This hypothesis is based on the observation that AAMø's express a number of genes known to be involved in cell proliferation and collagen synthesis, most prominent being the enzyme arginase-1 (Arg-1) [2],[9],[11],[12],[13],[14].

In contrast to iNOS, a widely investigated enzyme critically involved in many aspects of host immunity [15], much less is known about the role of arginase in infectious disease models [16]. Although it is known that arginases can antagonize NO synthesis by competing for L-arginine [17],[18],[19], the inducible Arg-1 isoform is believed to regulate other important functions as well. One of the major products of arginase is L-ornithine, a precursor in the production of polyamines and proline, which control cell proliferation and collagen production, respectively [16],[19]. It is thought that extracellular L-ornithine and L-proline, secreted from arginase expressing cells (AAMø's), are transported into fibroblasts, where they subsequently become incorporated into collagen [20]. Therefore, Arg-1 expressing cells have been hypothesized to be critical regulators of fibrosis. Thus, a better understanding of the mechanisms regulating Arg-1 activity could reveal novel strategies to control fibroproliferative diseases.

Since extracellular L-arginine is required for sustained NO and L-ornithine production [21], mechanisms controlling L-arginine transport may critically regulate iNOS and Arg-1 activity. Among the transport systems that facilitate L-arginine uptake, system y^+^ is considered to be the major L-arginine transporter in most cells and tissues [22]. Encoded by the solute carrier 7a1-3 (*Slc7a1-3*) family of genes, y^+^ is a Na+-independent high affinity amino acid transport system. CAT2 is the most dynamically regulated of the three transporters, with CAT1 operating as the product of a constitutively expressed “housekeeping” gene and CAT3 expressed primarily in the brain [23],[24]. Several pro-inflammatory mediators including LPS can regulate the expression of CAT2; thus, it likely functions as the key L-arginine transporter during inflammatory responses. Recent studies with CAT2-deficient mice showed sustained NO production in macrophages is dependent on CAT2 [25]. Thus, it appears to be the essential L-arginine transporter in macrophages. However, while CAT2 has been studied in the context of iNOS activity [25], no studies have addressed its role in the regulation of Arg-1 activity following infection.

To elucidate the function of the *Slc7a2* gene in vivo, we infected CAT2^−/−^ mice with either *Schistosoma mansoni* or *Toxoplasma gondii*; pathogens that induce highly polarized Th2 and Th1 responses, respectively [7]. Strikingly, following infection with *S. mansoni*, CAT2^−/−^ mice developed granulomas that were 3- to 4-times larger than WT and hepatic fibrosis (a feature of severe disease) was significantly exacerbated in chronically infected mice [26],[27],[28],[29], indicating a general worsening of Th2-associated pathologies in the absence of CAT2. The CAT2^−/−^ mice were also more susceptible to *T. gondii* infection, demonstrating that CAT2 is critical for the development of protective Th1-dependent immunity. Thus, these studies identify CAT2 as a powerful regulator of T_H_1 and T_H_2 effector responses, which may have major implications for a variety of infectious diseases.

## Results

### CAT2 suppresses pulmonary granuloma associated inflammation and fibrosis

**Figure 1:**
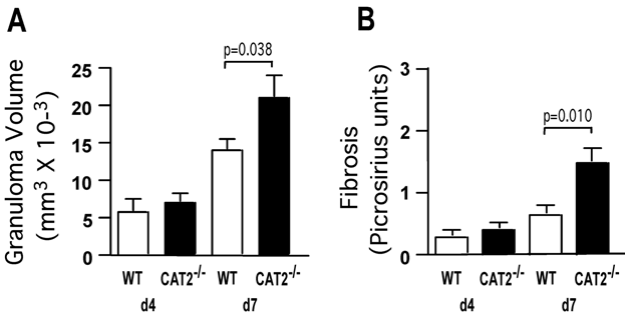
CAT2^−/−^ mice develop larger pulmonary granulomas and fibrosis is increased. WT C57BL/6 (N = 5) and CAT2^−/−^ (N = 5) mice were sensitized i.p. with *S. mansoni* eggs and then challenged 2 weeks later i.v. with 5000 live eggs. Mice were sacrificed on days 4 and 7 post-challenge and granuloma volume (A) and fibrosis (B) was quantified from Giemsa (A) and Picrosirius Red (B) stained lung sections. Statistically significant differences are shown. The experiment was repeated a total of three times with similar results.

To determine whether CAT2 plays a regulatory role during an acute Th2 response, we exploited the *S. mansoni* pulmonary granuloma model [30]. In this model, schistosome eggs are delivered to the lungs of mice via tail vein injection. The eggs are deposited in the pulmonary vasculature where they induce an eosinophil rich, CD4^+^ Th2 cell-dependent granulomatous response [7]. Wild-type (WT) and CAT2^−/−^ mice were sensitized and challenged with 5000 live *S. mansoni* eggs and on day 4 and 7 post-challenge, animals were sacrificed and the effects of CAT2 deficiency were examined microscopically in the lung. Although there were no significant differences in granuloma size or composition on day 4, the CAT2^−/−^ mice displayed an average 37% increase in granuloma size on day 7 (Fig. 1A), the peak of the granulomatous response [31]. The increase in peak granuloma size was also associated with significant picrosirius red staining of histological sections (Fig. 1B), providing evidence of increased fibrosis in the CAT2^−/−^ lung.

### Liver granuloma formation is exacerbated in *S. mansoni* infected CAT2^−/−^ mice

**Figure 2:**
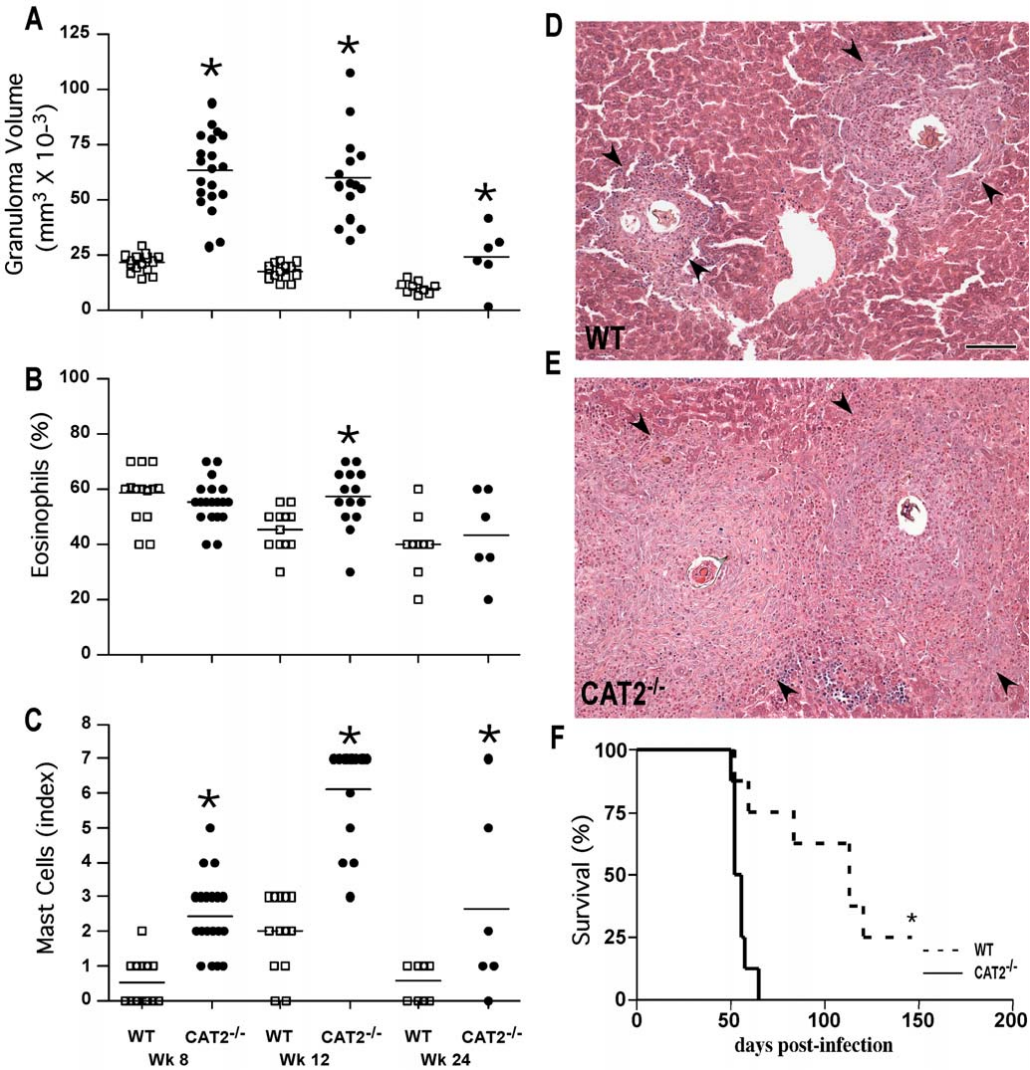
CAT2^−/−^ mice display increased granulomatous inflammation in the liver following infection with *S. mansoni.* WT C57BL/6 (open squares) and CAT2^−/−^ (filled circles) mice were infected with 30–35 *S. mansoni* cercariae and sacrificed on wks 8, 12 and 24 post-infection. The results shown are for individual mice pooled from 3 separate experiments. A. Liver granuloma volumes measured microscopically at 8, 12 and 24 wks post-infection. The * symbol denotes significant differences between WT and KO mice at that time point, p<0.05. B. Percentage of liver granuloma-associated eosinophils (% of total cells). C. Liver granuloma-associated mast cells (Scale 1–8). D. Representative granulomas from an infected WT C57BL/6 mouse (wk 8 post-infection). Arrows indicate the perimeter of an individual granuloma with the miracidium containing egg in the center. The bar in panel 2D = 200 microns. E. Representative granulomas from an infected CAT2^−/−^ mouse. F. % Survival after infection with 100 cercariae. p<0.001.

To explore the role of CAT2 during a chronic Th2-driven inflammatory response, WT and CAT2^−/−^ mice were infected with *S. mansoni* cercariae and the granulomatous response was examined in the liver 8, 12, and 24 weeks post-infection. As expected, peak granuloma size was observed at the acute time point (wk 8), with subsequent down-modulation in granuloma formation in chronically infected (wk 12–24) animals (Fig. 2A). When the responses in WT and CAT2^−/−^ mice were compared, however, it was clear that the CAT2^−/−^ mice developed granulomas 2- to 3-times larger than WT at all time points (Fig. 2A). There was also a small but significant increase in tissue eosinophils on wk 12 (Fig. 2B) and a consistent increase in mast cells in the CAT2^−/−^ granulomas (Fig. 2C). The representative photomicrographs shown in panels 2D (WT) and 2E (CAT2^−/−^) illustrate the exacerbated inflammatory response in the CAT2^−/−^ liver. When infected with a high dose of parasites, the CAT2^−/−^ mice also succumbed significantly faster than WT animals (Fig. 2F). However, at low doses, the survival of CAT2^−/−^ was not significantly different from WT through at least 24 wk of infection (not shown).

### The progression of liver fibrosis is accelerated in *S. mansoni* infected CAT2^−/−^ mice

**Figure 3:**
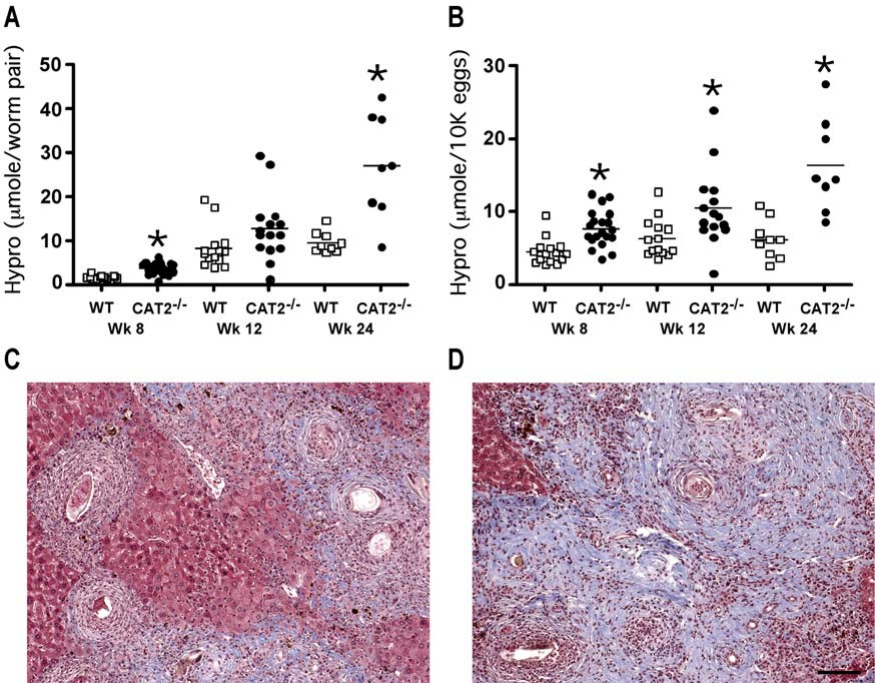
*S. mansoni* infected CAT2^−/−^ mice develop exacerbated liver fibrosis. WT C57BL/6 (open squares) and CAT2^−/−^ (filled circles) mice were infected with 30–35 *S. mansoni* cercariae and sacrificed on wk 8, 12 and 24 post-infection. A. Liver Fibrosis adjusted per worm pair (µmol of hydroxyproline/worm pair). The * symbol denotes significant differences between WT and KO mice at that time point, p<0.05. B. Liver Fibrosis per 10,000 eggs (µmol of hydroxyproline/1×10^4^ eggs). C. Representative liver granulomas stained with Masson's Trichrome (8 wk infected C57BL/6 mouse). D. Representative liver granulomas stained with Masson's Trichrome (8 wk infected CAT2^−/−^ mouse). The bar in panel 3D = 200 microns.

Liver fibrosis is the primary cause of chronic morbidity in *S. mansoni* infections [32]. To determine whether CAT2 regulates tissue fibrogenesis, liver tissue was taken at various time points post-infection and collagen content was measured as hydroxyproline [33]. Although both groups developed significant fibrosis, hydroxyproline levels were markedly increased in the CAT2^−/−^ livers, particularly at the chronic time points (Fig. 3A, 3B). Collagen deposition was also examined histologically with Masson's trichrome (Figs. 3C and 3D) and picrosirius red stains (not shown), and thick bands of collagen were seen throughout the livers of the infected CAT2^−/−^ mice. In contrast, collagen deposition was primarily in areas surrounding the granulomas in WT animals.

**Figure 4:**
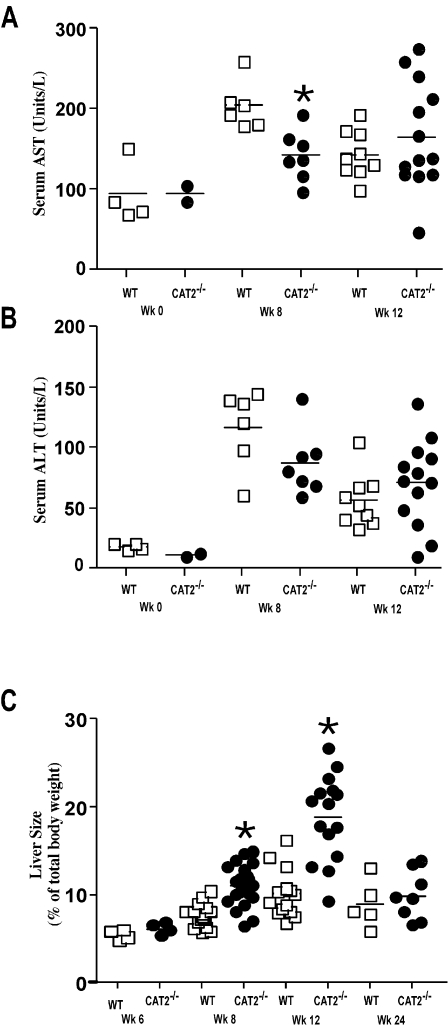
*S. mansoni* infected CAT2^−/−^ mice develop marked hepatomegaly. A. Serum aspartate transaminase (AST) values in WT C57BL/6 and CAT2^−/−^ mice (wk 0, 8, 12 post-infection). The * symbol denotes significant differences between WT and KO mice at that time point, p<0.05. B. Serum alanine transaminase (ALT) values. C. Liver size as percentage of body weight at 6, 8, 12, and 24 wk post-*S. mansoni* infection.

Serum AST (SGOT) and ALT (SGPT) levels were similarly increased in both groups following infection (Fig. 4A, 4B), indicating there was no evidence of significant egg-induced hepatotoxicity in the CAT2^−/−^ animals. In fact, AST/ALT levels were slightly reduced in the CAT2^−/−^ mice at the 8 wk time point. However, the CAT2^−/−^ mice displayed significant hepatomegaly, particularly at the acute and early chronic time points (Fig. 4C). There was also marked splenomegaly in the absence of CAT2 (data not shown). Thus, in contrast to the enhanced liver toxicity observed in IL-4^−/−^, IL-4Rα^−/−^, and LysMCreIL-4R^−/−^ mice [10],[29],[34], CAT2^−/−^ mice developed significant liver fibrosis, portal hypertension, and collateral vessels, which are features of severe hepatosplenic disease.

Importantly, the increased pathological responses in the CAT2^−/−^ mice were not attributed to differences in parasite burden since similar numbers of eggs and paired adult parasites were found in the tissues of both groups at all time points (Table S1).

### Th2 cytokine production is impaired in CAT2^−/−^ mice

**Figure 5:**
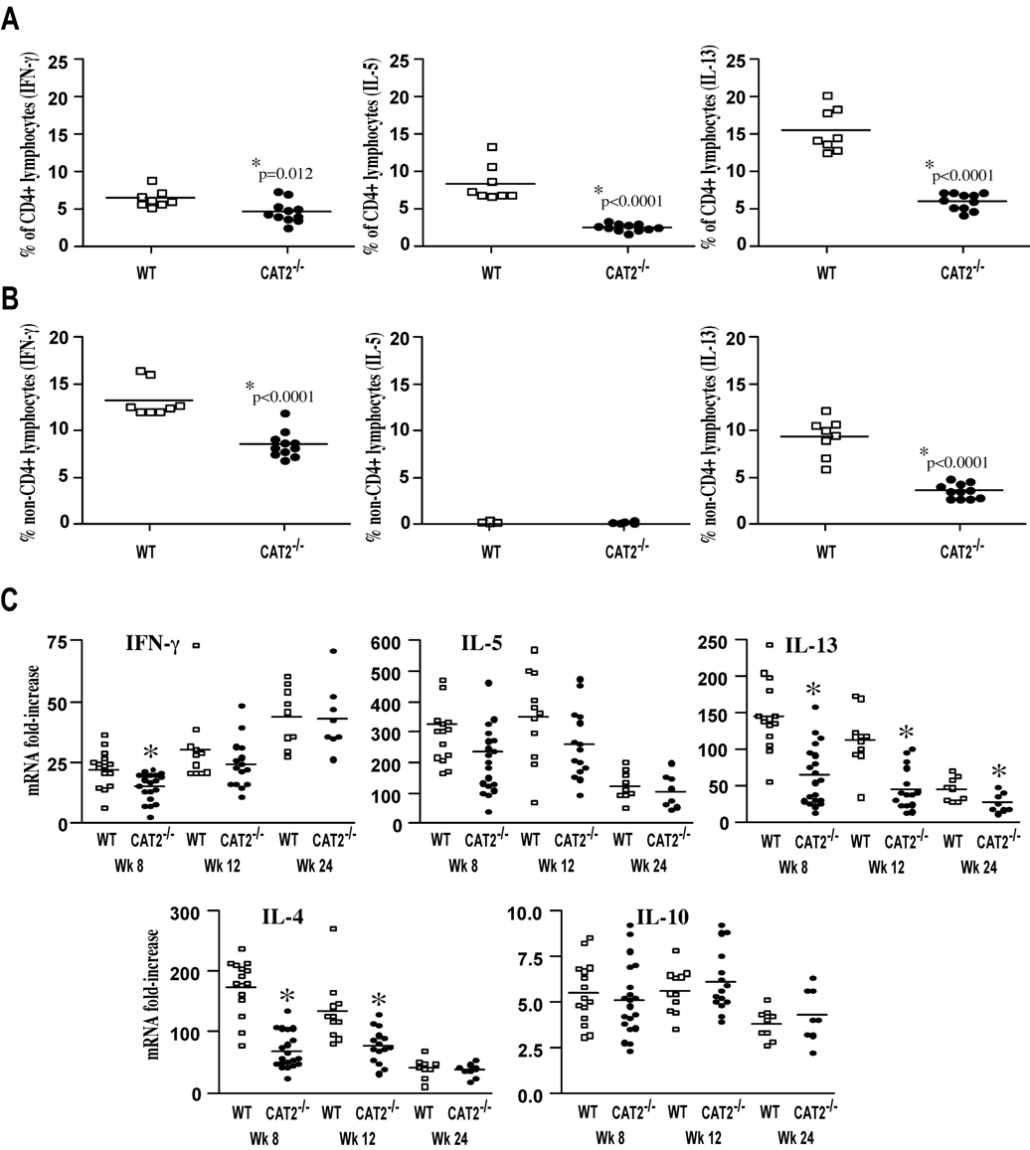
T_H_1/T_H_2 cytokine responses are reduced in infected CAT2^−/−^ mice. A. Leukocytes derived from the granulomatous livers of 8 wk *S. mansoni* infected mice were stimulated with PMA/Ionomycin/BFA for 3 hr and then stained for CD4, IFN-γ, IL-5 and IL-13. The data show the percentage of CD4^+^ lymphocytes producing a specific cytokine. Results for individual mice are shown and statistically significant differences are indicated in the figure. B. Frequency of non-CD4^+^ lymphocytes producing specific cytokines. C. Relative mRNA levels of *ifn-γ*, *il-5*, *il-13*, *il-4*, *il-10* in *S. mansoni* infected liver tissue were determined by real-time Q-RT-PCR (wk 8,12 and 24), by normalizing mRNA levels to expressed, endogenous HPRT mRNA. Fold increases in relative mRNA levels were determined by the ratio of normalized infected mRNA to uninfected mRNAs. Results from individual mice are shown. The * symbol denotes significant differences between WT and KO mice at that time point, p<0.05.

Granuloma formation and fibrosis are tightly controlled by the egg-induced Th2 response [5],[7]. Therefore, to determine whether local or systemic changes in Th2 cytokine production were responsible for the severe pathological reactions in CAT2^−/−^ mice, granuloma-associated lymphocytes were isolated from the livers of individual mice (wk 8) and IL-5, IL-13, and IFN-γ production was assayed by intracellular cytokine staining (ICS). Surprisingly, despite displaying a significant increase in pathology, the frequency of IL-5 and IL-13-producing CD4^+^ T cells was markedly reduced in the livers of infected CAT2^−/−^ mice (Fig. 5A). IL-13 production was also reduced in the non-CD4^+^ T cell population (Fig. 5B). The reduction in type-2 cytokines did not result from an increased type-1 response because the frequency of IFN-γ producing cells (CD4^+^ and CD4^−^ cells) was also reduced in the CAT2^−/−^ livers, but not to the same magnitude as the type-2 cytokine producing cells.

We also isolated RNA from the liver and examined IFN-γ, IL-5, IL-13, IL-4, and IL-10 mRNA responses by real-time PCR at 8, 12, and 24 wk (Fig. 5C). As expected [35], there was a marked increase in IL-4, IL-5, IL-10, IL-13, and IFN-γ mRNA in the livers of infected WT mice. However, consistent with the ICS results, IL-13 mRNA levels were significantly reduced in the CAT2^−/−^ mice at all time points post-infection. Similar results were seen with IL-4, although IL-5 mRNA was only slightly reduced in the knockout mice. Also consistent with the ICS studies, IFN-γ mRNA expression was reduced in the CAT2^−/−^ liver, but only significantly at the 8 wk time point. In contrast to the other cytokines, IL-10 mRNA levels increased to a similar extent in both groups at all time points post-infection. Together, these results indicate that CAT2 expression ensures maximal development of Th2 cytokine responses *in vivo*.

### Proliferation of granuloma-associated effector T cells is impaired in CAT2^−/−^ mice

**Figure 6:**
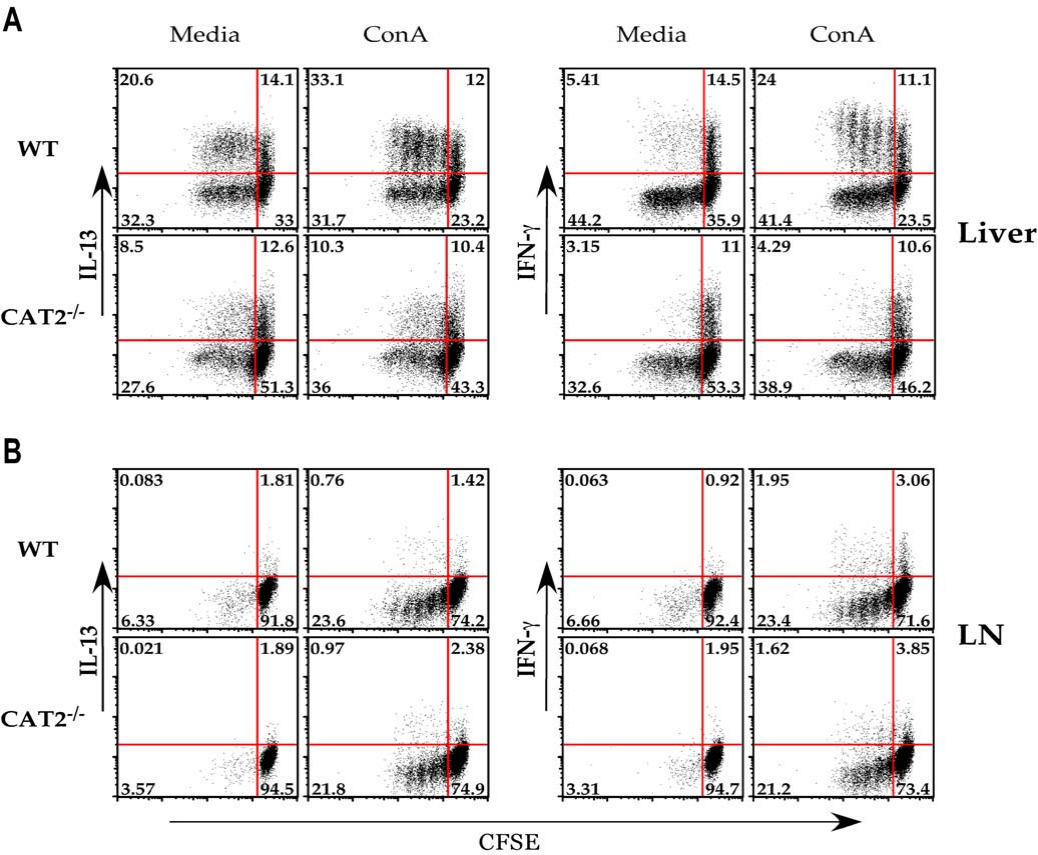
Cytokine production and proliferation of CD4^+^ lymphocytes is impaired in the granulomatous tissues of infected CAT2^−/−^ mice. A. Leukocytes derived from the granulomatous livers of 8 wk infected WT C57BL/6 and CAT2^−/−^ mice were stained with CFSE and cultured for 72 hrs in the presence of media alone or Con A (1 µg/ml). Cells were subsequently stained for IL-13 (left panels) and IFN-γ (right panels). Representative FACS plots are shown. The experiment was repeated numerous times with similar results. B. Mesenteric lymph node cultures from 8 wk *S. mansoni* infected WT and CAT2^−/−^ mice.

To explore mechanisms by which CAT2 regulates Th2 response development *in vivo*, we investigated whether the proliferation of cytokine-producing cells was affected by CAT2 deficiency. Purified lymphocytes isolated from the granulomatous livers (Fig. 6A) and mesenteric lymph nodes (Fig. 6B) were CFSE-labeled and stimulated polyclonally with ConA for 72 hr. Following stimulation, cells were assayed by intracellular cytokine staining for IFN-γ and IL-13, as markers of Th1 and Th2 effector cells, respectively. In the liver (Fig 6A), there was significant proliferation without additional ConA stimulation, indicating the presence of a large population of antigen-activated T cells in the granulomatous tissues of both WT and CAT2^−/−^ mice. 34.7% of the lymphocytes in the unstimulated WT group were also producing IL-13, which increased to 45.1% after Con A stimulation. The majority of the cytokine producing cells were also proliferating (20.6% before and 33.1% after Con A stimulation), indicating the presence of a large population of effector Th2 cells in the infected WT liver. In contrast, only 21.1% of the CAT2^−/−^ lymphocytes were producing IL-13 and no increase was observed after Con A stimulation. The CAT2^−/−^ IL-13-producing cells were also proliferating at a much slower pace (8.5% before and 10.3% after ConA stimulation). Similar results were seen for IFN-γ (right panels), although in general there were more IL-13 than IFN-γ producers in the liver. The number of proliferating IFN-γ producing cells in WT liver was 24%, which decreased to less than 5% in CAT2^−/−^ mice (Con A stimulated), demonstrating that both the proliferative and cytokine producing capabilities of granuloma-associated lymphocytes were diminished in the absence of CAT2. As expected, the frequency of cytokine producing cells was much lower in the MLN (Fig. 6B). Moreover, although the frequency of cytokine-producing cells increased following Con A treatment, there were no significant differences between the two groups, suggesting that the impaired cytokine and proliferative responses of CAT2^−/−^ mice were restricted to the granulomatous tissues.

In addition to Th1/Th2 cytokines, we also examined whether FoxP3, IL-17, and TGF-β1 expression were altered in the infected CAT2^−/−^ mice. In contrast to the marked effect observed on Th2 cytokine expression, however, granuloma-associated CD4^+^ T cells from CAT2^−/−^ and WT mice displayed similar IL-17, FoxP3, and TGF-β1 responses. In fact, the Th17 response was weak when compared with the Th2 cytokine response. For example, at 9 wk post-infection, the percentage of CD4^+^ T cells that were IL-13 positive was 17.1% and 11.8% in WT and CAT2^−/−^ mice, respectively, while only 0.19% and 0.2% were IL-17 positive. Although we observed significant FoxP3 expression in the liver, the responses in WT and CAT2^−/−^ were again nearly identical, with 6.87% of WT and 6.19% of CAT2^−/−^ CD4^+^ T cells expressing FoxP3. There were also no significant difference in TGF-β1 mRNA expression in the livers of infected WT and CAT2^−/−^ mice (not shown).

### Arginase activity is increased in CAT2^−/−^ alternatively-activated macrophages

**Figure 7:**
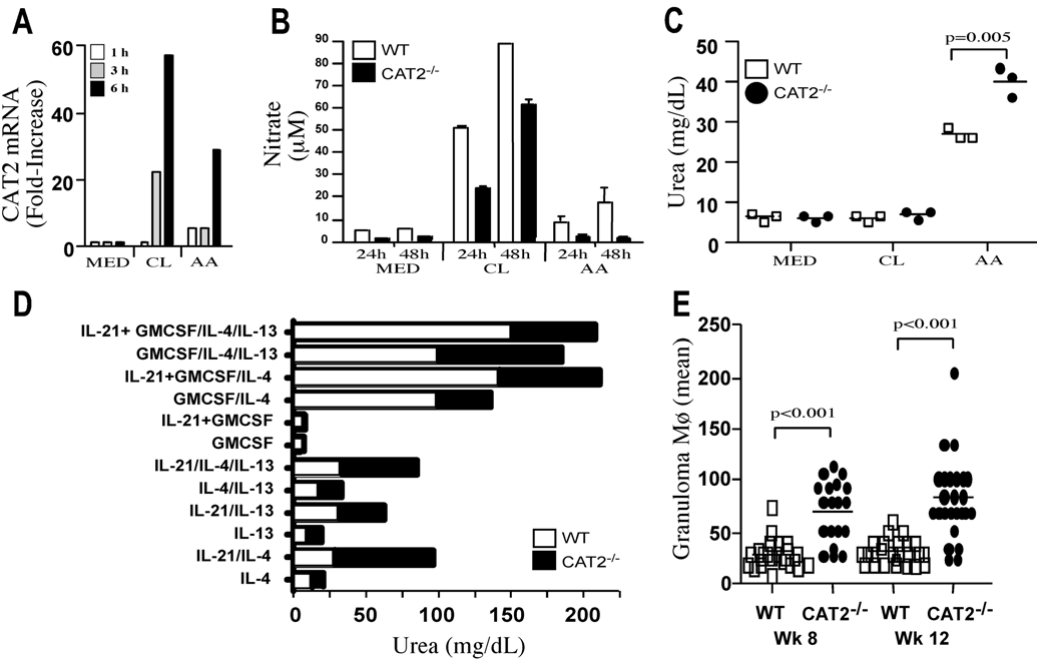
CAT2^−/−^ BMMφ display increased arginase activity but reduced NO responses. A. Messenger RNA for CAT2 is induced in BMMφ following classical (CL) or alternative activation (AA). BMMφ were incubated with medium alone (MED), IFN-γ/LPS (100 U/ml/100 ng/ml, CL), or IL-13/IL-4 (20 ng/ml, AA) for 1–6 hrs. Total RNA was isolated from individual wells and CAT2 expression was analyzed by RT-PCR. The supernatants were also collected. B. NO expression by activated CAT2^−/−^ BMMφ is impaired following classical (IFN- γ/LPS) or alternative activation (IL-13/IL-4). Supernatants were assayed for nitrate levels 24 and 48 hr post-stimulation. C. Urea production (a measure of arginase activity) was assessed in WT and CAT2^−/−^ BMMφ's following stimulation with CL or AA stimuli. D. CAT2^−/−^ BMMφ display increased arginase activity when exposed to multiple “alternative” activating stimuli. WT and CAT2^−/−^ macrophages were stimulated with various combinations of IL-21, GM-CSF, IL-4 and IL-13 (all 20 ng/ml). E. Macrophages in granulomas at 8 and 12 wk post-infection with *S. mansoni* (% adjusted proportionally by granuloma size). Spots represent the mean of 30 granulomas analyzed per mouse.

Numerous studies have demonstrated that granuloma formation and hepatic fibrosis are dependent on Th2 cytokines [26],[29],[36]; therefore, it was surprising to find a markedly reduced Th2 cytokine response in the granulomatous tissues of the CAT2^−/−^ mice, since immunopathology increased significantly in these animals. Because Arg-1 and iNOS activities are regulated by the availability of L-arginine [17] and alternatively-activated macrophages play an important role in the pathogenesis of schistosomiasis [9],[10], we examined whether CAT2 deficiency was regulating the function of alternatively-(AA) or classically- (CL)-activated macrophages. CAT2 mRNA levels were increased 20- to 40-fold in both classically and alternatively activated macrophages, suggesting that CAT2 activity is not restricted to a Th1- or Th2-polarized response (Fig. 7A). CAT2 mRNA levels were also increased over 10-fold in the granulomatous tissues of infected mice (data not shown). Interestingly, however, when nitric oxide and urea levels (a quantitative measure of arginase activity) were measured, the NO producing ability of macrophages was decreased in the absence of CAT2, regardless of the activation stimuli used (Fig. 7B). In marked contrast, urea production was significantly increased in alternatively-activated CAT2^−/−^ macrophages (Fig. 7C). IL-4, IL-13, IL-21, and GM-CSF have all been shown to increase arginase activity in macrophages [13],[37],[38]. Interestingly, the CAT2^−/−^ macrophages displayed enhanced arginase activity with nearly every stimulus examined (Fig. 7D). Finally, there were also significantly more macrophages in the CAT2^−/−^ granulomas (Fig 7E), suggesting that the increase in granuloma size was due in part to the increased recruitment of macrophages to the liver.

### Fibroblast arginase activity is increased in the absence of CAT2

**Figure 8:**
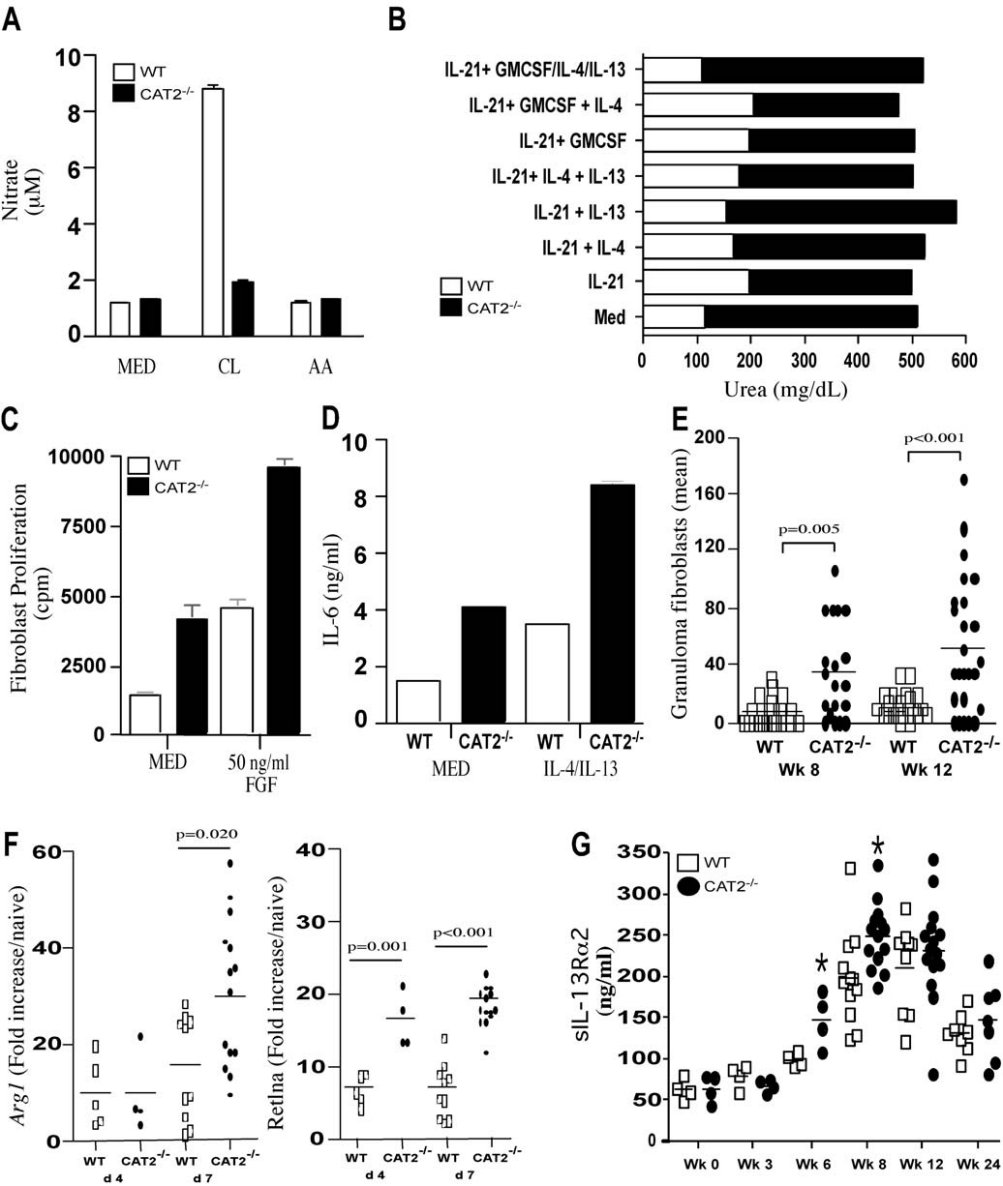
CAT2^−/−^ primary fibroblasts display increased proliferative responses and spontaneous arginase activity. A. WT C57BL/6 (open bars) and CAT2^−/−^ (filled bars) primary lung fibroblasts were stimulated with medium alone, IFN-γ/LPS (100 U/ml/100 ng/ml, CL), or with IL-4/IL-13 (20 ng/ml, AA). After 24 hr, NO activity was quantified by measuring nitrate levels in the supernatants. B. WT and CAT2^−/−^ fibroblasts were plated at 5 × 10^5^ cells per well and stimulated with various type-2 cytokines that promote alternative activation. Arginase activity was quantified by measuring urea production (mg/dL). C. WT and CAT2^−/−^ fibroblasts were plated at 1 × 10^5^ cells/well, stimulated with medium alone or 50 ng/ml FGFb, and proliferation was measured by (H^3^) thymidine incorporation. D. WT and CAT2^−/−^ fibroblasts were plated at 1 × 10^5^ cells/well and cultured in the presence of medium alone or IL-4/IL-13 (20 ng/ml). After 24 hr incubation, the supernatants were assayed for IL-6. All assays were repeated with similar results. E. Fibroblasts in granulomas at 8 and 12 wk post-infection with *S. mansoni* (% adjusted proportionally by granuloma size). Spots represent the mean of 30 granulomas analyzed per mouse. F. WT and CAT2^−/−^ were sensitized with 5000 live *S. mansoni* eggs i.p. and then challenged 2 wk later with 5000 eggs i.v. On day 4 and 7 post-challenge, lung tissue was harvested and *Arg1* and *Retlna* mRNA expression was quantified by real-time PCR and graphed as fold-increase over naïve lung. The results for individual mice are shown. G. WT C57BL/6 (open squares) and CAT2^−/−^ (filled circles) mice were infected with *S. mansoni* cercariae and serum was collected at various time points post-infection. Serum IL-13Rα2 levels were measured by ELISA in individual mice. The * symbol denotes significant differences between WT and KO mice at that time point, p<0.05.

**Figure 9:**
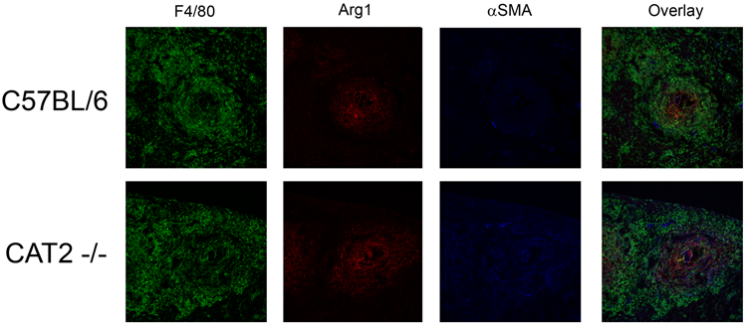
Arg-1 and α-SMA expression colocalized in CAT2^−/−^ liver granulomas. C57BL/6 and CAT2^−/−^ mice were infected percutaneously with *S. mansoni* for 10 weeks. Portions of livers were removed and frozen. 8 µM liver sections from C57BL/6 and CAT2^−/−^ mice were stained anti-F4/80-Alexa 488 (Green), anti-Arg1-Alexa 647(Red), and anti-Alpha Smooth Muscle Actin-Texas Red (Blue). Individual 20× images were taken for each channel and then combined to provide a composite image. Images are representative of 3 individual mice from independent experiments.

Next we investigated fibroblast activity. For these studies, primary fibroblasts were generated from lung tissue and in initial studies, the production of NO and urea was compared in WT and CAT2^−/−^ fibroblasts following classical or alternative activation. In contrast to classically activated macrophages, where NO expression was only partly CAT2 dependent (Fig. 7B), production of NO by CAT2^−/−^ fibroblasts was almost entirely dependent on CAT2 activity (Fig. 8A). Nevertheless, the amount of NO produced by CL-activated fibroblasts was nearly ten-fold lower than macrophages plated at the same density (Figs 7B and 8A). Unlike macrophages, in which IFN-γ/LPS was strictly required for iNOS activity and IL-4/IL-13 for arginase activity (Fig. 7B and 7C), we observed significant spontaneous arginase activity in primary fibroblasts. Indeed, unstimulated WT fibroblasts (Fig. 8B) produced nearly the same amount of urea as alternatively-activated WT macrophages (Fig. 7C). There was also no evidence of enhanced arginase activity in fibroblasts following stimulation with IL-4, IL-13, IL-21, or GM-CSF (Fig. 8B). Most striking however, was the 4- to 5-fold increase in arginase activity in the CAT2^−/−^ fibroblasts. The CAT2^−/−^ fibroblasts also proliferated more rapidly, both spontaneously and in response to FGF stimulation (Fig. 8C). In addition, production of IL-6, a key cytokine in fibroblast proliferation and activation was also increased in the CAT2^−/−^ fibroblasts, both at baseline and in response to IL-4/IL-13 stimulation (Fig. 8D). Consistent with these in vitro observations, we detected significantly more fibroblasts in CAT2^−/−^ liver granulomas at both 8 and 12 wk post-infection (Fig. 8E). Finally, to provide evidence that alternative activation was increased in vivo, we injected WT and CAT2^−/−^ mice intravenously with 5000 viable *S. mansoni* eggs and examined the expression of Arg1 and Retlna (RELM-α/Fizz1) mRNA in the lung at 4 and 7 days post-injection. As shown in Figure 8F, both genes associated with alternative activation were significantly upregulated in the CAT2^−/−^ lung. Finally, we also stained liver sections from infected mice with antibodies to Arg1, alpha smooth muscle actin (α-SMA), and F4/80, to characterize the pattern of Arg1 expression in vivo. Consistent with the enhanced fibroblast activity observed in vitro, the CAT2^−/−^ granulomas showed much greater staining for α-SMA, a marker of activated myofibroblasts. They also displayed much stronger staining for Arg1 and the overlay (purple staining) suggested that the majority of Arg1 was associated with myofibroblasts, with lesser staining observed in macrophages (Fig. 9).

### Hepatic fibrosis in infected CAT2^−/−^ mice is IL-13 independent

The IL-13 receptor alpha 2 functions as a decoy receptor for IL-13 [39], and studies conducted with IL-13Rα2^−/−^ mice demonstrated that the decoy receptor inhibits the development of hepatic fibrosis in schistosomiasis [40],[41]. Because fibroblasts are believed to be the key producers of sIL-13Rα2 and fibroblast function was altered in the absence of CAT2 (Fig. 8), we measured the circulating levels of IL-13Rα2 in infected CAT2^−/−^ mice, since changes in IL-13Rα2 expression might be contributing to their exacerbated IL-13-associated pathologies. Surprisingly however, we found either similar, or at some time points, increased levels of sIL-13Rα2 in the infected CAT2^−/−^ mice (Fig. 8G). Thus, despite displaying decreased IL-13 responses (Figs. 5–6) and increased IL-13 decoy receptor levels (Fig. 8G), the CAT2^−/−^ mice developed an exacerbated fibrotic response.

**Figure 10:**
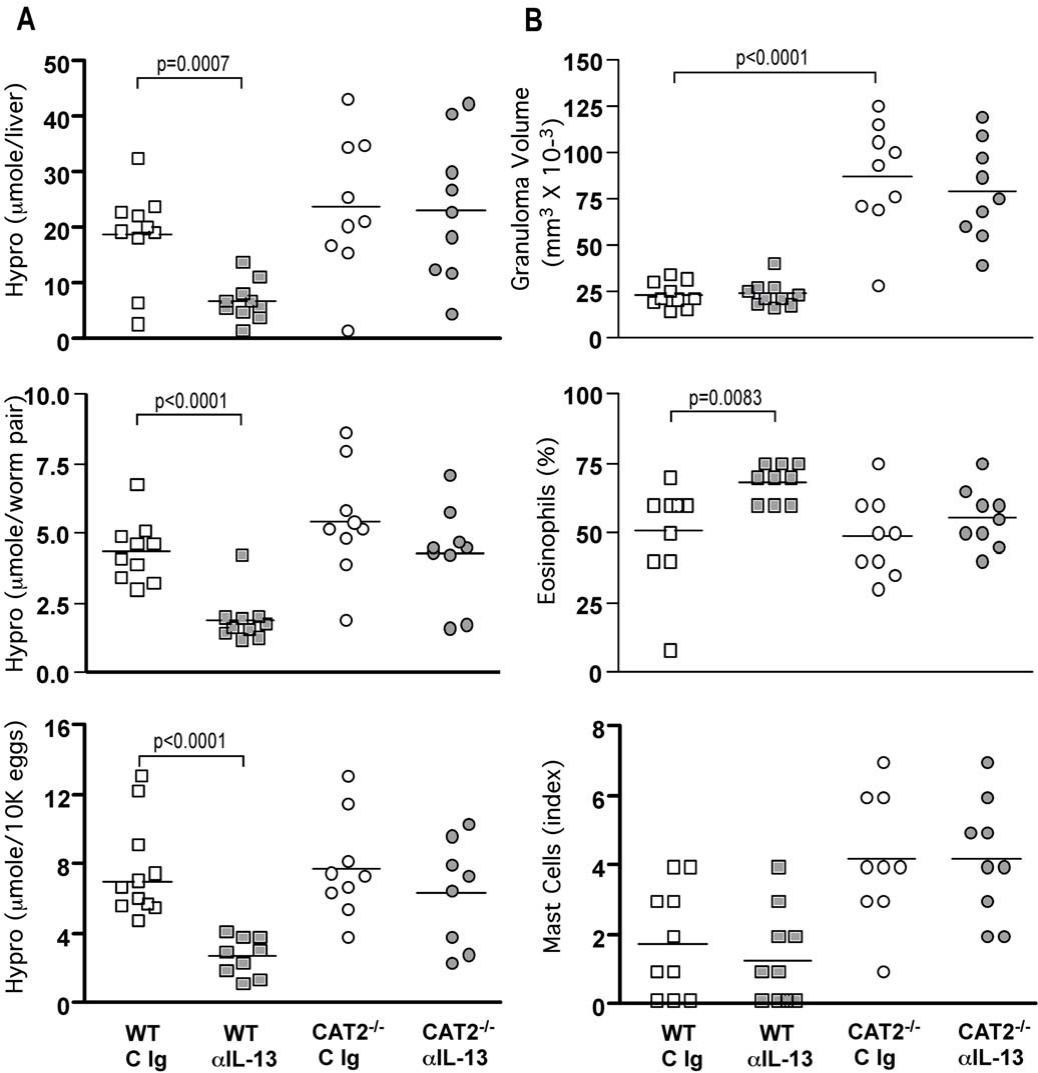
Development of hepatic fibrosis in infected CAT2^−/−^ mice is IL-13-independent. WT C57BL/6 (squares) and CAT2^−/−^ (circles) mice were infected with 30–35 cercariae. Separate groups were treated with either control antibody (C Ig, open symbols) or with a neutralizing mAb to IL-13 (α-IL-13, filled symbols) weekly for a total of 4 wk starting on wk 5 post-infection. The animals were sacrificed on wk 9 and fibrosis and granuloma formation were assessed. A. Liver hydroxyproline levels in individual mice, shown as µmol/liver, µmol/worm pair, and µmol/10K eggs. Significant differences are indicated in each figure. B. Granuloma size (volume, mm^3^ × 10^−3^), percentage of granuloma-associated eosinophils, and mast cell indices were determined microscopically.

Next, to determine whether the severe liver pathology in the infected CAT2^−/−^ mice was in fact dependent on IL-13, we infected WT and CAT2^−/−^ mice with *S. mansoni* and inhibited IL-13 with a neutralizing mAb. As expected [26], IL-13 blockade significantly decreased fibrosis in WT mice (Fig. 10A) without affecting the overall magnitude of the granulomatous response (Fig. 10B). Surprisingly however, IL-13 blockade was completely ineffective in CAT2^−/−^ mice (Fig. 10A), suggesting that their fibrotic response was independent of IL-13 activity. Importantly, similar numbers of eggs and paired adult parasites were found in the tissues of all groups (Table S2).

### CAT2 augments Th1-dependent immunity by regulating nitric oxide production

**Figure 11:**
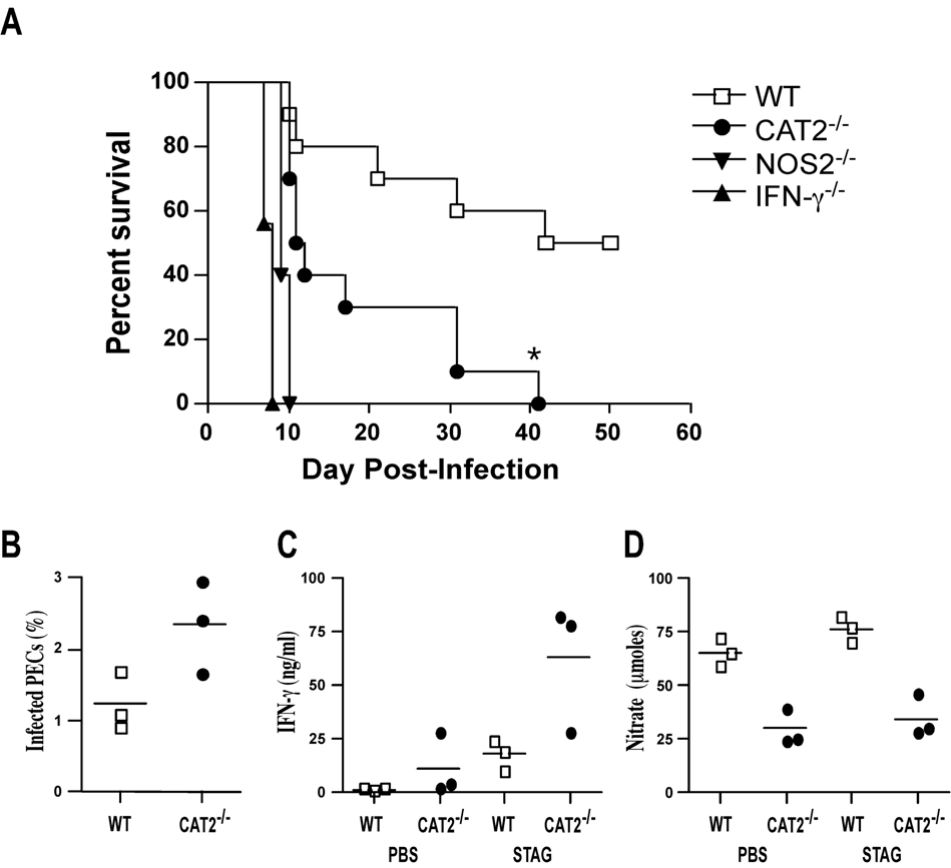
Development of Th1 immunity is compromised in CAT2^−/−^ mice. A. WT C57BL/6 (open squares), CAT2^−/−^ (filled circles), NOS2^−/−^ (filled inverted triangles) and IFN-γ^−/−^ (filled triangles) mice were infected i.p. with 20 *T. gondii* cysts, and survival was assessed up to 50 days post-infection. (N = 5/group). B. Peritoneal exudate cells (PECs) were prepared from WT and CAT2^−/−^ mice on day 7 post-infection. The percentage of infected cells was determined microscopically by evaluating a minimum of 700 cells per slide (3 mice/group). C. PECs were placed in culture for 24–48 hr and either left untreated or restimulated with STAG. Culture supernatants were collected and IFN-γ levels were determined by ELISA. D. NO activity was evaluated in the same culture supernatants by measuring nitrate production.

In a final series of experiments, we investigated whether CAT2 is required for the development of Th1-dependent immunity, since NO production was impaired in CAT2^−/−^ macrophages (Fig. 7B), as well as in fibroblasts (Fig. 8A). In these studies, the *Toxoplasma gondii* model was used, since resistance is known to be mediated by an IFN-γ and NO-dependent mechanism [42]. Initially, we examined whether susceptibility was altered in the CAT2^−/−^ mice by monitoring host survival following infection with *T. gondii*. IFN-γ^−/−^ and NOS2^−/−^ mice were included as controls. As expected, WT mice were much more resistant than either IFN-γ^−/−^ and NOS2^−/−^ mice, with approximately 50% of the WT animals surviving through day 50 (Fig. 11A). In contrast, 100% of the IFN-γ^−/−^ and NOS2^−/−^ mice succumbed between days 7–10 post-infection, while CAT2^−/−^ animals displayed an intermediate phenotype, with 100% mortality observed by day 42. We also infected WT and CAT2^−/−^ mice and isolated peritoneal exudate cells (PECs) on day 7 to quantify the number of infected cells and to examine IFN-γ and NO responses *ex vivo*. Consistent with their enhanced susceptibility, the percentage of infected cells increased in the CAT2^−/−^ mice (Fig. 11B). This was also associated with a significant increase in IFN-γ production, both at baseline and following stimulation with soluble *T. gondii* antigen (STAG). Nevertheless, despite displaying much stronger IFN-γ responses, production of NO was markedly decreased in the CAT2^−/−^ PECs, which likely explains their enhanced susceptibility.

## Discussion

Previous studies have suggested that CAT2 controls NOS2 and arginase activity by regulating arginine transport into cells [43], however the relative importance of CAT2 in the development of Th1 and Th2 effector functions was not investigated. Here, we examined the role of CAT2 encoded by the *Slc7a2* gene in vivo by studying two well-established infectious disease models, characterized by the development of either protective Th1- or pathogenic Th2-type immune responses [5],[42]. We found that CAT2-deficient mice were significantly more susceptible to the Th1-inducing pathogen *T. gondii*. The increased susceptibility was attributed to the attenuated NO response, which led to uncontrolled parasite replication. When CAT2^−/−^ mice were challenged with the Th2-inducing pathogen *S. mansoni*, the animals developed significantly worse Th2-associated pathology, despite displaying weaker Th2 responses. Importantly, the pathological changes in the CAT2^−/−^ mice were associated with increased arginase activity in fibroblasts and alternatively activated macrophages. These results reveal an essential role for CAT2 in the development of Th1 immunity. However, they also suggest that CAT2 functions as a potent negative regulator of Th2-associated pathology, most likely by limiting arginase activity in important effector cells like fibroblasts and macrophages.

NO production by iNOS contributes to normal cellular processes, resistance to intracellular pathogens, and pathophysiological conditions [23],[44]. MacLeod and colleagues found that CAT2 is induced coordinately with iNOS in numerous cell types and studies conducted with CAT2-deficient cells demonstrated that arginine uptake via CAT2 is required for sustained NO production in macrophages [25] and to a lesser extent in astrocytes [45]. However, NO synthesis in fibroblasts was only partially dependent on CAT2, suggesting that other compensating transporters can provide arginine for iNOS-mediated NO synthesis [46]. Thus, the dependence on CAT2-mediated L-arginine transport for NO production appears to vary in different cell types. Moreover, the relative importance of CAT2 in the development of NO-dependent immunity in vivo was previously unknown.

To evaluate the function of CAT2 in vivo, we infected CAT2^−/−^ mice with the intracellular pathogen *T. gondii*. Resistance to *T. gondii* is mediated by an IFN-γ and NOS2-dependent mechanism [42]. Therefore, we compared CAT2^−/−^ mice with IFN-γ- and NOS2-deficient animals, since they are known to rapidly succumb to *T. gondii* infection. Interestingly, despite developing a significantly stronger IFN-γ response (due to the higher parasite burdens), the CAT2^−/−^ mice were much more susceptible to *T. gondii*, with all animals succumbing within 6 weeks. The increased susceptibility was associated with a markedly attenuated NO response, suggesting that CAT2 is critically important to the development of Th1-associated immunity. However, the fact that NOS2^−/−^ mice died earlier than the CAT2^−/−^ animals suggests that NO synthesis in vivo is only partly dependent on CAT2. This was consistent with the reduced but not completely ablated NO responses of CAT2^−/−^ peritoneal exudate cells.

Since NOS2 and Arg-1 both require L-arginine as a substrate [1], we hypothesized that CAT2 might also regulate important Th2 effector functions. Recent in vitro studies with bone marrow-derived macrophages demonstrated that CAT2 is induced by both Th1- and Th2-type stimuli [43],[47], which was consistent with our observations. Moreover, studies conducted with macrophages showed that L-arginine transport is significantly impaired in the absence of CAT2, regardless of the stimuli used to activate the cells [47]. Thus, it was suggested that CAT2 regulates both the classical and alternative activation of macrophages [43]. Because Th2-driven alternative macrophage activation plays a critical role in the pathogenesis of schistosomiasis [9],[10], we investigated the function of the CAT2 gene in the murine model of schistosomiasis. Strikingly, although CAT2 deficiency did not affect the establishment of *S. mansoni* infection, Th2-associated pathology in the liver was exacerbated and the animals died at a significantly accelerated rate when compared with WT mice. Indeed, granuloma size increased more than 3-fold and development of hepatic fibrosis was exacerbated. Similar results were also obtained with the *S. mansoni* pulmonary granuloma model.

Because granuloma formation and fibrosis are driven by the Th2 cytokine response [7],[10],[26],[27],[29],[35],[36],[48], we initially examined whether CAT2^−/−^ mice were developing stronger Th2 responses. Unexpectedly, despite displaying a significant increase in Th2-associated pathology [6], the frequency of cytokine-producing CD4^+^ Th2 cells was markedly reduced in the livers of the infected CAT2^−/−^ mice (Fig. 5A). The granuloma associated CD4^+^ Th2 lymphocytes also proliferated less when restimulated in vitro. Together, these results indicate that CAT2 is required for the maximal development of Th2 responses. Thus, the severe pathological changes in the CAT2^−/−^ mice were paradoxically associated with reduced rather than enhanced Th2 cytokine production.

Previous studies with IL-4Rα^−/−^ and some IL-4-deficient mice demonstrated that development of the Th2 response is critical for survival in schistosomiasis, especially during the early stages of infection [10],[27],[49],[50]. In addition, recent studies with macrophage/neutrophil-specific IL-4Rα-deficient mice suggested that the development of alternatively activated macrophages, in particular, is critically important for host survival. [10]. Despite developing significantly weaker Th2 responses, however, the CAT2^−/−^ mice showed no signs of increased susceptibility to *S. mansoni* when infected with a low dose of parasites, with all of the knockout animals successfully establishing chronic infections. Moreover, in contrast to infected IL-4Rα and LysM^(Cre)^IL-4Rα^(−/flox)^ animals [10], the CAT2^−/−^ mice did not default to a Th1-type immune response. They also displayed no evidence of significant hepatoxicity as determined by their serum AST/ALT responses. In fact, liver enzymes were slightly reduced in the CAT2^−/−^ mice when compared with infected WT animals. These data, when combined with the histological findings discussed above, suggest that alternative macrophage activation is not significantly impaired in the infected CAT2^−/−^ mice. In fact, evidence was obtained both in vitro and in vivo that alternative activation increased in the absence of CAT2.

To investigate this hypothesis further, we stimulated WT and CAT2^−/−^ bone marrow-derived macrophages with cytokines that are known to promote alternative macrophage activation including, IL-4, IL-13, IL-21, and GM-CSF [13],[37],[38] and examined the induction of arginase activity, a key feature of AAMøs [1],[51],[52]. As expected, the Th2-associated cytokines triggered significant arginase activity in WT macrophages. However, the macrophages generated from CAT2^−/−^ mice consistently displayed a markedly exaggerated response. These data demonstrate that CAT2 functions as a negative regulator of arginase activity in macrophages, which may in part explain their exacerbated fibrotic response. Thus, although it was recently suggested that CAT2 could regulate both the classical and alternative activation of macrophages [43], our combined in vitro and in vivo data indicate that the primary role of CAT2 is to optimize NO production in classically-activated macrophages, while limiting arginase activity in alternatively-activated cells. The maintenance of arginine transport by the constitutive arginine transporter CAT1 could explain the preservation of arginase activity in the CAT2^−/−^ mice.

Although alternatively-activated macrophages are believed to be important regulators of wound healing and fibrosis [1],[5], fibroblasts are the primary collagen secreting cells. While it was previously shown that CAT2 has only minimal effect on NO production in classically-activated fibroblasts [46], the effects of CAT2-deficiency on arg1 activity was not examined. To determine whether CAT2 regulates fibroblast activation, we generated primary lung fibroblasts from WT and CAT2^−/−^ mice and stimulated the cells with various Th1 or Th2-type stimuli. Surprisingly, although classically-activated fibroblasts were in general less potent producers of NO than macrophages, we observed a significant (>75%) reduction in NO production in CAT2^−/−^ fibroblasts. In combination with earlier studies focused on embryonic fibroblasts that reported a minimal (<20%) effect on NO production [46] , our data suggest the dependence on CAT2 for NO synthesis varies in different fibroblast subpopulations.

We also examined the effects of CAT2 deficiency on arginase activity. In contrast to macrophages, however, where arginase activity was strictly dependent on Th2 cytokine stimulation, fibroblasts displayed no significant cytokine inducible arginase response, even when stimulated with an optimal combination of Th2-type cytokines [37],[38]. Nevertheless, when arginase activity in WT and CAT2^−/−^ fibroblasts was compared, the CAT2^−/−^ fibroblasts exhibited much greater arginase activity at baseline. The fibroblasts from CAT2^−/−^ mice also proliferated faster and produced significantly more of the autocrine growth factor IL-6 [53], both before and after stimulation with IL-4 and IL-13 [54],[55],[56]. Thus, the enhanced arginase activity in CAT2^−/−^ fibroblasts and AAMø's likely contributed to their exacerbated inflammatory and fibrotic responses following infection with *S. mansoni*. The increased arginase response may also explain the suppressed CD4^+^ Th2 cell responses in the granulomatous tissues. Because T cells, macrophages, and fibroblasts all compete for arginine, the increased arginase activity in CAT2^−/−^ macrophages and fibroblasts may have reduced arginine levels in the granulomatous tissues, resulting in the local suppression of CD4^+^ T cell responses, as has been postulated recently in related studies [17],[57],[58]. In contrast to classically activated macrophages, AAMø's are also known to be inefficient stimulators of T cell proliferation [59], with F4/80^+^ alternatively activated macrophages functioning as potent inhibitors of antigen-specific CD4^+^ T cell proliferative responses in vivo [58].

Fibroblasts are also important producers of the soluble IL-13Rα2 [40],[60], which can function as a decoy receptor for IL-13 and was recently shown to inhibit the development of fibrosis in schistosomiasis [39],[40],[41]. Since CAT2 fibroblasts displayed an unusual activated phenotype, we examined whether production of the sIL-13Rα2 was also altered in the CAT2^−/−^ mice. Decreased production of the sIL-13Rα2 could provide a simple and straightforward explanation for their exacerbated IL-13-driven pathological responses. Surprisingly, the exact opposite was observed. Indeed, serum levels of sIL-13Rα2 were either the same or slightly increased in the infected CAT2^−/−^ mice. Thus, we questioned whether the development of fibrosis in CAT2^−/−^ mice was in fact dependent on IL-13. To formally address this question, we performed a series of studies with neutralizing antibodies to IL-13 [61]. Strikingly, although IL-13 blockade had a highly significant anti-fibrotic effect in WT mice, CAT2^−/−^ mice were unresponsive. These observations, when combined with the reduced IL-13 and enhanced IL-13Rα2 responses, suggest that the development of fibrosis in CAT2^−/−^ mice is to a large extent IL-13-independent.

When viewed together, the data point to fibroblasts and AAMø's as the key mediators of the exacerbated pathological response, since arginase activity was increased in the CAT2^−/−^ cells. In the case of fibroblasts, the enhanced arginase response also appeared to be independent of Th2 cytokine stimulation. Arg1 and α-SMA expression also colocalized in the granulomatous livers and both proteins were expressed at much higher levels in the infected CAT2^−/−^ mice, confirming that there were more activated myofibroblasts. However, there was no evidence of spontaneous liver fibrosis in uninfected CAT2^−/−^ mice, suggesting that a chronic inflammatory stimulus or some type of tissue damage was needed to initiate the fibroproliferative response. Nevertheless, a recent study found that CAT2-deficient mice are susceptible to the development of spontaneous inflammation in the lung [62]. The same group also showed that CAT2 expression is linked with the development of asthma [63]. Thus, mucosal tissues, which are repeatedly exposed to irritants, may be particularly sensitive to changes in CAT2 activity. As such, CAT2 may be involved in the regulation of a wide variety of diseases that are normally associated with chronic Th2 responses.

In conclusion, these studies demonstrate for the first time that CAT2 is critically important for the development of IFN-γ/NO-dependent immunity to the intracellular protozoan pathogen *T. gondii*. In addition, by inhibiting arginase activity in fibroblasts and alternatively-activated macrophages, CAT2 functions as a powerful negative regulator of type-2 cytokine-driven pathology. Thus, these findings may have major implications for a wide variety of infectious and inflammatory diseases.

## Materials and Methods

### Mice, parasite infections, and antigen preparation

Female C57BL/6 were obtained from Taconic Farms (Germantown, NY) [64]. Breeding pairs of C57BL/6 CAT2^−/−^ mice were obtained from the UCSD Cancer Center (La Jolla, CA) [25]. Mice were housed under specific pathogen-free conditions at the National Institutes of Health in an American Association for the Accreditation of Laboratory Animal Care approved facility. The NIAID animal care and use committee approved all experimental procedures. *S. mansoni* eggs were extracted from the livers of infected mice (Biomedical Research Institute, Rockville, MD) as previously described [35]. For the induction of secondary granulomas, mice were sensitized intraperitoneally (i.p.) with 5000 live eggs, and then challenged with 5,000 live eggs i.v [31]. In the infection experiments, mice were infected percutaneously via the tail with 30–35 cercariae of a Puerto-Rican Strain of *S. mansoni* (NMRI) obtained from infected *Biomphalaria glabrata* snails (Biomedical Research Institute). Soluble egg Antigen (SEA) was obtained from purified and homogenized *S. mansoni* eggs [33]. All animals underwent perfusion at the time of sacrifice so that worm and tissue egg burdens could be determined [33]. 20 cysts of the avirulent ME49 strain were inoculated i.p into C57BL/6, CAT2^−/−^ , NOS2^−/−^ (Taconic), and IFN-γ^−/−^ (Taconic) mice for morbundity studies. In some studies, mice were sacrificed day 7 post-inoculation and PECs were harvested and set up in culture for 24 and 48 hours in media and with soluble tachyzoite Ag (Stag), which was prepared as described [65].

### Histopathology and fibrosis

The sizes of pulmonary and hepatic granulomas were determined on histological sections that were stained with Wright's Giemsa stain (Histopath of America, Clinton, MD). Approximately 30 granulomas per mouse were included in all analyses. A skilled pathologist evaluated the percentages of eosinophils, mast cells, and other types of cells in the same sections. The number of schistosome eggs in the liver and the gut and the collagen content of the liver, as measured by hydroxyproline levels, were determined as previously described [33]. Specifically, hepatic collagen was measure as hydroxyproline by the technique of Bergman and Loxley [66]. The increase in hepatic hydroxyproline was positively related to egg numbers in all experiments and hepatic collagen is reported as the increase above normal liver collagen in µmoles per 10,000 eggs; (infected liver collagen – normal liver collagen)/liver eggs × 10^−4^ or µmoles per worm pair. At late chronic time points, fibrosis is reported as total liver collagen per liver. The same individual scored all histological features and had no knowledge of the experimental design.

### Flow cytometry, cell isolation, proliferation assays, and ICC

Mesenteric lymph nodes (MLN) and about 200 mg of granulomatous liver tissue was disrupted into single cell suspension by grinding through a 100 µm nylon mesh. The WBCs from liver cells were separated on a 34% percoll gradient (350 g for 20 min) (Fluka). MLN and Liver WBCs were treated with 2 ml of ACK lysis buffer (Quality Biological) for 2 min. Purified leukocytes were stained with 5 mM CFSE (Molecular Probes) for 5 min at RT. Excess CFSE was quenched by washing the cells in RPMI supplemented with 10% FBS. 3×10^6^ cells were cultured in 24 well plates and were either left unstimulated or stimulated with 1 µg/ml of Con A for 72 hours. (note, the WBCs separated from the liver contain live *S. mansoni* eggs and therefore all liver leukocyte cultures are exposed to soluble egg antigens as well). *ICC*: Liver leukocytes either freshly isolated (ex vivo) or restimulated for 72 hrs were stimulated with PMA (10 ng/ml), Ionomycin (1 µg/ml) and BFA (10 µg/ml) (Sigma) for 3 hrs. Cells were surface stained for CD4 PE-Cy5 (BD Biosciences), fixed in 2% formaldehyde for 20 min at RT, permeabolized with 0.1% saponin buffer (Sigma) and stained for IFN-γ (APC or FITC), IL-5 APC (BD Biosciences) and IL-13 PE (Centocor) and acquired with FACS Calibur®. Data were analyzed in Flowjo® V8.

### RNA isolation and real time polymerase chain reaction

Lung and liver tissue samples were placed individually in 500 µl of RNAlater and frozen at −20°C (Ambion). Samples were removed from RNAlater and placed in 500 µl TRIzol reagent (Invitrogen) to purify RNA. Total RNA was further purified using RNeasy Mini Kit from Qiagen (Qiagen Sciences). RNA (1 µg) was reverse-transcribed using Superscript II (Invitrogen, Carlsbad, CA) and quantification of transcripts was performed using Applied Biosystems (Foster City, CA) pre-designed gene expression assays for IFN-γ, IL-5, IL-13, IL-4, and IL-10. Each Taqman assay was run in duplicate. For each sample 5 ul of a 1∶30 dilution of cDNA reaction cocktail in a 20 ul final volume TaqMan reaction was used for each assay. Reaction preparation and thermal cycling were carried out following the manufacturer's protocol with a modification of increasing qPCR cycles to 50. Assay samples were normalized to HPRT expression and compared to uninfected controls according to comparative C_T_ method (Applied Biosystems).

### Bone marrow derived macrophages

Bone marrow was recovered from female C57BL/6 and CAT2^−/−^ mice and cultured in Petri dishes (100 × 15 mm) containing supplemented DMEM media (20% L929 conditioned medium) for a period of 6 days. After six days cells were harvested and seeded at a concentration of 5 × 10^5^ cells/well in 24 well plates containing supplemented DMEM media (10% FBS, 2 mM L-glutamine, 100 U/mL penicillin, and 100 ug/mL streptomycin). Cells were stimulated for 16 hr with combinations of IL-4, IL-13, and GMCSF (20 ng/ml), IFNγ (100 U/mL), or LPS (100 ug/mL)( Peprotech). In some cases, the cells were pretreated with IL-21 (R&D) for a period of 6 hr. Supernatants were collected for NO analysis and cells were lysed for arginase activity and RNA isolations. Real-time RT-PCR was performed on an ABI PRISM 7900HT Sequence Detection System (Applied Biosystems). Relative quantities of mRNA was determined using SYBR Green PCR Master Mix (Applied Biosystems) and by the comparative threshold cycle method. In this method, mRNA levels for each sample were normalized to hypoxanthine guanine phosphoribosyl transferase (HPRT) mRNA levels and then expressed as a relative increase or decrease compared with levels in media only controls. Primers were designed using Primer Express software (version 2.0; Applied Biosystems). Primers for CAT2-F CTC CTG GGT GCT CTG AAC CA and CAT2-R CTT CTC CCC TCC CGT TGA AC.

### Primary lung fibroblasts

Whole lungs were harvested in cRPMI supplemented with 10% FBS (Hyclone), 2 mM- L-Glutamine, 100 µg/ml penicillin–streptomycin (Gibco), 50 uM ß-mercaptoethanol (Sigma), minced into small pieces, and exposed to Collagenase D (1 mg/ml) (Roche) and 4 U/ml DNase I (Sigma) for 40 mins at 37°C with shaking. Tissues were disrupted by straining through a 100 micron nylon mesh (BD Falcon). The single cell suspensions were plated in Iscove's Modified Dulbecco's Medium with 2 mM L-glutamine, 5% FCS, 25 mM HEPES, 100 µg/mL Streptomycin, 100 U/mL Penicillin, 50 uM 2-ME. (3 lungs plated on 3–100 × 15 mm Petri dishes). 50% of media was changed on day 7 and cells were recovered on day 14 by adding 4 mL of HyQtase (Hyclone) reagent for 20 min and rigorously pipetting repeatedly to remove cells. Cells were then cultured at 5 × 10^5^ cells/well in 24 well plates. After activation with cytokines, supernatants were collected for NO analysis and IL-6 determination. Other cells were lysed to determine arginase activity or cultured for fibroblast proliferation.

### Enzyme-Linked Immunosorbent Assay (ELISA)

*IL-13Ra2* levels were determined by ELISA as previously described [41]. The concentration of IL-13Rα2 in the sample was determined from a serial-fold diluted standard of rmIL-13Rα2 Fc/chimera (R & D Systems). The sensitivity of the assay was approx. 98 pg/ml. IL-6 levels were measured using murine IL-6 DuoSet ELISA Development System (R&D Systems) according to the manufacturer's protocol. IFN-γ was assayed by sandwich ELISA, as previously described [41], and quantitated by comparison with standard curves generated with rIFN-γ (provided by Genentech, San Francisco, CA, and Genetics Institute, Cambridge, MA, respectively).

### Nitrite analysis and arginase assays

The concentration of nitrite in supernatants of primary lung fibroblasts and bone-marrow derived macrophages stimulated *in vitro* was determined spectrophotometrically by using the Griess reagent. Supernatants were collected after 16 hours, mixed 1/1 with Griess reagent, and absorbance measured at 543 nm using a SpectraMax 190 (Molecular Devices, Sunnyvale, CA). The nitrite concentration was determined using sodium nitrite as standard. In the arginase assays, cells were plated at 5 × 10^5^ per well in 96 well tissue culture plates and stimulated with combinations of IL-4, IL-13, and IL-21 (20 ng/mL). IL-21 was added 6 hours prior to IL-4 or IL-13 stimulation. Following stimulation, cells were washed with PBS and lysed with 0.1% TritonX-100 containing protease inhibitor (Roche). Lysates were transferred into a 96 well PCR plate and incubated with 10 mM MnCl_2_ and 50 mM Tris HCl (pH 7.5) to activate enzyme for 10 min at 55°C. After enzyme activation, 25 µl of lysate was removed and added to 25 µl 0.5 M arginine (pH 9.7) in a new PCR plate and incubated for 1–2 hours at 37 C. 5 µl of each sample was added in duplicate to a 96 well ELISA plate along with 5 µl of each standard, diluted in same assay conditions, starting at 100 mg/dL. Urea determination reagent from BioAssay Systems Quantichrome Urea Assay Kit was used according to the manufacturer's protocol.

### IL-13 blocking experiments

C57BL/6 (10/group) mice were infected percutaneously via the tail with 30–35 *S. mansoni* cercariae. Beginning on wk 5 post-infection, mice were treated with either mouse anti-IL-13 mAb [61] or GL113 control antibody (Harlan Bioproducts). Each mouse received one 0.5 mg dose/wk via i.p. injection on wk 5, 6, 7 and 8. Mice were sacrificed on wk 9 post-infection.

### *T. gondii* infection studies

Acute tachyzoite growth was assessed using cytocentrifuge smears of peritoneal cells as previously described [65]. Differential analyses, including assessment of intracellular *T. gondii* infection, were performed on 700 or more cells per animal. Single-cell suspensions were prepared from peritoneal exudates and washed in CRPMI. Peritoneal cells were cultured at 2.5 × 10^6^ cells/mL in 200 µl of RPMI 1640 supplemented with 10% FBS, penicillin (100 U/ml), streptomycin (100 mg/ml), L-glutamine (2 mM), HEPES (10 mM), and 2-ME (Sigma) in the presence or absence of soluble tachyzoite Ag (5 µg/ml). Supernatants were harvested 24 and 48 hr later for determination of levels of IFN-γ and NO.

### Cell proliferation assays

WT and CAT2^−/−^ primary lung fibroblasts (1 × 10^5^/well) were plated in Iscove's Modified Dulbecco's Media in 96 well flat bottom plates (BD Falcon) for 16 hr at 37°C. 1 µCi/well of [^3^H]thymidine (Amersham) was added for 24–72 hrs. Cells were frozen at −20 C and were later collected onto a glass fiber filter pads (LKB Wallac, Turku, Finland) using a 96-well harvester (Tomtec, Orange, CT). Scintillation cocktail was (XSC/9200; LKB Wallac) added, and radioactivity determined on a liquid scintillation counter (Betaplate model 1205, LKB Wallac). Some cells were stimulated with FGFβ at 50 ng/ml (Invitrogen). Each sample was set up in triplicate.

### Fluorescent imaging and microscopy

8 um liver sections were taken from WT and CAT2^−/−^ 10 wk *S. mansoni* infected livers. Sections were fixed in cold acetone for 10 minutes and stored at −20°C. Slides were washed 3× in DPBS and incubated with F4/80 conjugated with Alexa 488 (Caltag Clone CI:A3-1), Alpha Smooth Muscle Actin (Sigma-Aldrich Clone 1A4) conjugated with Texas Red, and Arginase1 (Santa Cruz Biotechnology Clone V-20) conjugated with Alexa 647. Images were collected on a Leica SP5 confocal microscope (Leica Microsystems, Exton, PA USA) using a 20× oil immersion objective NA 0.70. Fluorochromes were excited using an Argon laser for Alexa 488, an Orange Helium-Neon laser for Alexa 594 and a Red Helium-Neon laser for Alexa 647. To avoid possible crosstalk the wavelengths were collected separately and later merged. Images were processed using Leica LAS-AF software (version 1.7.0 build 1111).

### Statistics

Hepatic fibrosis (adjusted for egg number) decreases with increasing intensity of infection (worm pairs). Therefore, these variables were compared by analysis of covariance, using the logarithm of total liver eggs as the covariate and the logarithm of hydroxyproline content per egg. Variables that did not change with infection intensity were compared by one-way ANOVA or Student's *t* test [33]. Changes in cytokine mRNA expression and granuloma size were evaluated using ANOVA. Differences were considered significant when p<0.05.

## Supporting Information

Table S1 Worm and Egg Burdens
(0.04 MB DOC)

Table S2 Worm and Egg Burdens
(0.03 MB DOC)
