# Supplementary material for: Cationic Amino Acid Transporter-2 Regulates Immunity by Modulating Arginase Activity
Source: PLoS Pathog. 2008 Mar 14;4(3):e1000023. doi: 10.1371/journal.ppat.1000023 (PMC2265428; doi:10.1371/journal.ppat.1000023)
Supplement: Table S1 — Worm and Egg Burdens (0.04 MB DOC) [file ppat.1000023.s001.doc]

| Week  Postinfection | Group | Worm Pairs | Total Worms | Eggs/worm pair  (in thousands) |
| --- | --- | --- | --- | --- |
| 9 | WT (n=15) | 3.93 + 0.65 | 9.53 + 1.25 | 5.43 + 0.47 |
| 9 | CAT2^-/-^ (n=20) | 4.40 + 0.37 | 10.15 + 0.78 | 6.14 + 0.44 |
| 12 | WT (n=14) | 3.00 + 0.47 | 7.29 + 1.08 | 14.18 + 1.13 |
| 12 | CAT2^-/-^ (n=15) | 4.06 + 0.53 | 9.13 + 0.99 | 13.31 + 1.19 |
| 24 | WT (n=9) | 2.89 + 0.42 | 6.44 + 0.78 | 18.53 + 2.85 |
| 24 | CAT2^-/-^ (n=8) | 2.13 + 0.44 | 4.75 + 0.92 | 18.96 + 2.74 |

**Table 1. Worm and Egg Burdens**
