# Supplementary material for: Cationic Amino Acid Transporter-2 Regulates Immunity by Modulating Arginase Activity
Source: PLoS Pathog. 2008 Mar 14;4(3):e1000023. doi: 10.1371/journal.ppat.1000023 (PMC2265428; doi:10.1371/journal.ppat.1000023)
Supplement: Table S2 — Worm and Egg Burdens (0.03 MB DOC) [file ppat.1000023.s002.doc]

| Group | Treatment | Worm Pairs | Total Worms | Eggs/worm pair  (in thousands) |
| --- | --- | --- | --- | --- |
| WT (n=10) | Control Ig | 4.60 + 0.71 | 12.10 + 1.28 | 7.26 + 0.40 |
| WT (n=10) | Anti-IL-13 | 4.10 + 0.62 | 10.40 + 1.37 | 7.49 + 0.95 |
| CAT2^-/-^ (n=9) | Control Ig | 4.67 + 0.91 | 12.60 + 1.57 | 8.63 + 0.99 |
| CAT2^-/-^ (n=9) | Anti-IL-13 | 5.44 + 0.65 | 13.20 + 0.99 | 7.37 + 0.80 |

**Table 2. Worm and Egg Burdens**
